# Influence of Multi-Walled Carbon Nanotubes on the Mechanical and Deformation Performance of Polymer-Modified Crumb Rubber Concrete

**DOI:** 10.3390/polym18040503

**Published:** 2026-02-17

**Authors:** Arveendh A/l Vasudevan, Bashar S. Mohammed, Naraindas Bheel

**Affiliations:** 1 Civil and Environmental Engineering Department, Universiti Teknologi PETRONAS, Seri Iskandar 32610, Malaysia; 2 Department of Civil Engineering, AROR University of Art, Architecture, Design and Heritage Sukkur, Rohri 65170, Sindh, Pakistan

**Keywords:** crumb rubber, multiwalled carbon nanotubes, concrete, hardened concrete, RSM modelling and optimization

## Abstract

Crumb rubber (CR), a recycled elastomeric polymer derived from scrap tyres, has been used as a partial replacement for fine aggregates in concrete to manage non-biodegradable waste tyre piling, which fills landfills and harms the environment. Polymer-modified rubber improves the concrete’s flexibility, toughness, and impact resistance, but reduces its strength and modulus of elasticity. Multi-walled carbon nanotubes (MWCNTs) are being used to mitigate these issues. The purpose of this study is to investigate the impact of CR% (1% to 5%) as a partial replacement for sand by volume and MWCNTs (at a percentage of 0.05% to 0.08%) as additives by weight of cement as input parameters for determining the mechanical strength (compressive, tensile, and flexural) and deformation properties (modulus of elasticity and Poisson’s ratio) of MWCNT- and polymer-modified CR concrete using response surface methodology (RSM). The results show that 0.05% MWCNT and 1% CR content led to increases in compressive strength, flexural strength, and tensile strength by 14.12%, 11%, and 13.68%, respectively. In addition, models to predict those properties have been developed using RSM with a 95% reliability level. It has been observed that the notable development in the mechanical characteristics of CR concrete with the accumulation of MWCNTs and the models constructed using RSM were deemed satisfactory, with a variation of 0.05% to 0.065% of MWCNTs along with 2% CR.

## 1. Introduction

Urbanization and industrialization are driving a surge in transportation demand, resulting in increased vehicle production and an eventual rapid increase in waste tyres globally [1]. Annually, 1 billion waste tyres are produced, with over half discarded without pre-treatment, and this is expected to increase to 1.2 billion by 2030 [2], posing environmental and health risks [1,3]. Non-biodegradable scrap tyres, if landfilled for long time, release toxic substances that percolate through soil, contaminating the water table and eventually waterways [4].

Another issue associated with waste tyres is their high flammability due to their calorific properties, which makes them problematic to extinguish, causing black smoke, higher temperatures, air pollution, and global warming by releasing additional CO_2_ into the atmosphere [5]. Improperly discarded waste tyres, due to their concave shape, provide a favourable environment for the proliferation of various pests, including rats, mosquitoes, and reptiles. This, in turn, heightens the likelihood of the transmittance and growth dangerous illnesses such as Zika virus, dengue fever, and malaria [5]. To efficiently handle discarded tyres, they are primarily utilized in the manufacturing of tyre-derived fuels via burning, the creation of carbon black, and as fuels for cement furnaces. Nevertheless, the primary drawback of tyre-derived gasoline is its significant release of CO_2_ into the atmosphere during manufacturing. It is less cost-effective when compared to fuel obtained through petroleum resources [2]. Additionally, CR is applied for terrestrial purposes, including as bitumen for constructing roads and motorways, surfaces for sporting facilities, bedding for animals, and as a filler product for turf grasses [6,7]. The building engineering sector in the United States only uses a small portion of the available CR—about 7% as of 2015 [8].

The construction sector has an adverse impact on environmental sustainability, as it consumes a significant quantity of natural resources, including aggregates. River sand, which is commonly utilized as a fine aggregate, is decreasing swiftly and is becoming limited and costly, owing to excessive consumption. To address the environmental concerns and disposal challenges of waste tyres, researchers have utilized CR particles from waste tyres as a partial replacement for sand in concrete production [9].

Researchers have shown that incorporating CR into concrete might negatively impact its strength due to the insufficient bonding between the hardened cement paste and the CR particles’ surface [10]. Loganathan et al. [11] displayed a reduction of 44.83% in strength while using 5% CR compared to the control mixture. Mohammed et al. [12] described the use of CR in concrete, causing strength reductions. Gamil et al. [13] detailed that the incorporation of CR into concrete caused a decline in strength. According to prior studies [14,15,16,17], the strength of the concrete reduces by 0.7% to 79% when the amount of CR in the concrete increases. Inadequate bonding may also result from the inclusion of zinc stearate in the tyre manufacturing process, which subsequently permeates the rubber surface when mixed with concrete, causing the CR to retain air on its surface and repel water [18]. This leads to an increasing air volume in the fresh mix, resulting in a larger void capacity and increased porosity in the hardened concrete. As a consequence, the durability, strength, and stiffness of the concrete are reduced [12,18]. Furthermore, the presence of entrapped air on the CR surface contributes to an increase in the thickness of the ITZ, which consequently leads to poor bonding with the hardened matrix and ultimately leads to a decrease in strengths and immature failures. Hence, to ensure the efficient utilization of CR in concrete, it is essential to minimize the reduction in strength [17,19,20,21]. To develop the bonding between hardened cement paste and CR, several studies have investigated the inclusion of nanoparticles in concrete [22,23,24,25].

An investigation has been conducted into the influence of including carbon nanotubes (CNTs) in cement-based ingredients. The impact depends upon several factors, such as the specific characteristics of the nanotube, its dimensions, the treatment it undergoes (functionalization), and the technique used. Achieving even dispersion of CNTs in concrete mixtures requires specialized procedures. Rashad [26] stated that the dispersal of CNTs in a matrix is a challenging owing to the agglomeration of particles caused by van der Waals interactions in the solution. Various methods have been used to hinder the clustering of CNT, such as ultrasonic dispersers [27,28,29], magnetized stirrers [28,30], acidic functionalization of CNTs [30], and ultrasonic waves, which are the most prevalent indications of dispersing CNTs [31]. However, it should be noted that ultrasonic waves alone are not adequate to efficiently stop the clustering of CNTs [32]. To enhance efficiency, several chemical ingredients are included in the combination to aid in the dispersal of CNTs. For example, acetone is utilized in conjunction with multiple surfactants [28,29,33,34,35]. Appropriately disseminated CNTs employed in concrete have multiple advantages. CNTs have the capability to decrease pores, thereby enhancing mechanical characteristics [28,36]. Therefore, this study explores the potential of incorporating CR as a fine aggregate and MWCNT in concrete, which could transform sustainable building approaches and improve the mechanical characteristics of concrete. Crumb rubber, produced from recycled tires, provides an environmentally sustainable substitute for traditional fine aggregates, mitigating the problem of tire waste and enhancing resource recycling. Nonetheless, the use of crumb rubber alone may diminish the mechanical performance of concrete owing to its lower strength and inadequate adhesion with cement. The use of MWCNTs, recognized for their remarkable strength, toughness, and conductivity, may enhance the structural integrity and mechanical characteristics of concrete, as well as its durability. Looking into how CR and MWCNTs work together could lead to new types of concrete composites that combine sustainability with performance. This could reduce the need for natural aggregates, improve the properties of the material, and promote the development of high-performance, eco-friendly concrete.

This study is crucial for promoting sustainable building methods and providing resilient, economical solutions for contemporary infrastructure. Prior investigations on the combined impact of CR and multi-walled carbon nanotubes (MWCNTs) in concrete when using RSM modelling have been very limited. Currently, there is a need for scientific and numerical assessment of the mechanical and deformation characteristics of concrete with different CR and MWCNTs. This investigation has been achieved through the usage of multi-objective response surface methodology (RSM) modelling and optimisation. This research aimed to examine the mechanical and deformation characteristics of concrete. Additionally, it sought to explore the potential use of MWCNTs and CR in concrete.

## 2. Experimental Programme

### 2.1. Materials

**Figure 1:**
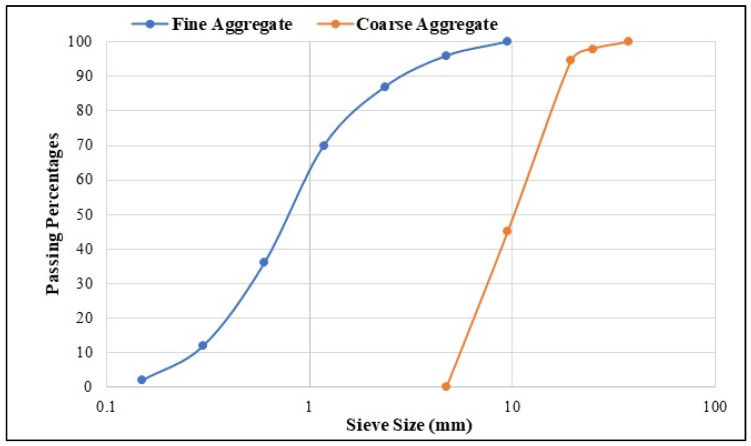
Sieve analysis curve for FA and CA.

The materials utilized in this research work were Portland Cement (PC) in agreement with the necessities of ASTM C150 [37], and its chemical composition is shown in Table 1. Multiwalled carbon nanotubes (MWCNTs) were manufactured by Nanocyl S.A. (Sambreville, Belgium) and used as additive by mass of PC. The properties of MWCNTs are revealed in Table 2. In addition, river sand with a specific gravity of 2.64 passed through a 4.75 mm sieve was used as the fine aggregate, and for the coarse aggregate (CA), river sand with a specific gravity of 2.66 passed through a 20 mm sieve. These aggregates were acquired from the local market in Perak, Malaysia. The sieve analysis of aggregates was carried out in agreement with ASTM C136 [38], as shown in Figure 1, while the specific gravity for sand and coarse aggregates was determined by obeying the ASTM C128 [39] and ASTM C127 [40]. Crumb rubber (CR) with a specific gravity of 0.92 and which passed through a 1.18 mm sieve was used in this research work. Furthermore, a polycarboxylate-based superplasticizer (SP) with the specific gravity and pH value of about 1.08, and 6.20, respectively, was added to the rubberized concrete to increase the workability of the mixture.

### 2.2. Pre-Treatment Method for CR

**Figure 2:**
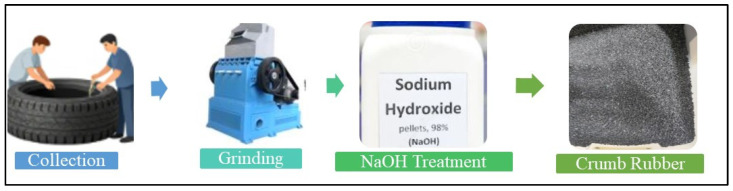
Pre-treatment process for CR.

The pre-treatment procedure for CR prior to its incorporation in concrete entails many stages to improve its performance and suitability within the concrete mixture [42]. Initially, crumb rubber, sourced from discarded tires, is purified to eliminate impurities such as metals, fibres, or dirt. This is accompanied by physical or chemical treatments to enhance its bonding characteristics with cement. A prevalent chemical treatment involves surface modification with sodium hydroxide (NaOH) or alternative chemical substances that decrease the hydrophobic characteristics of rubber and improve its adherence to the cement matrix. The pre-treatment process is shown in Figure 2.

### 2.3. Design Mix Using RSM Modelling

The RSM examines the relationships between input parameters and one or more responses (output parameters). It can predict the model for the output variables, providing an accurate model even with limited information from experiments. This approach is often used when numerous variables affect the outcome. Two variables were used in RSM: CR as a substitute for sand by volume (1–5%) and MWCNTs as an additive in terms of the mass of cement (0.05% to 0.08%). Table 3 shows 13 testing runs developed with the CCD approach of RSM, with repeated combinations to ensure accuracy and reduce fluctuations [43]. The RSM assesses how input relationships affect results. The assessed parameters involved mechanical and deformation characteristics. In all mixes, the amount of water was kept constant at 202 kg/m^3^.

### 2.4. Sample Preparation and Testing Procedures

#### 2.4.1. Mixing of Rubberised Concrete Combined with MWCNTS

**Figure 3:**
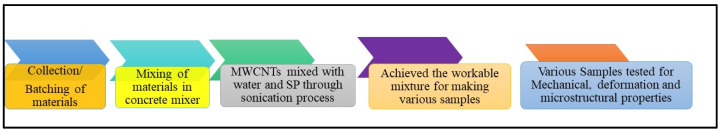
Mixing process of CR concrete combined with MWCNTs.

The preparation, batching and mixing of materials was carried out using BS 1881: Part 125:1986 standards [44]. The cement, aggregates, and CR were carefully combined for a period of 2 min in a mixer. Afterward, water was mixed with MWCNTs and superplasticizer (SP) to efficiently disperse the MWCNTs through a sonication process, and mixing continued for another 5 min. The efficacy of dispersing was essentially evaluated via the uniformity of fresh-state characteristics and the reproducibility of mechanical test outcomes, while microstructural examinations further validated an enhanced and comparatively uniform distribution at reduced MWCNT concentrations. The agglomeration seen at greater doses is ascribed to enhanced van der Waals forces, which restrict the efficacy of sonication after a particular concentration. The SP was added to counterbalance the reduction in fluidity and attain a workable mixture. Then, the samples for each test were prepared and left in the lab for 24 h, and then demoulded and put in a curing water tank for 28 days. The mixing process of the CR concrete combined with MWCNTs is indicated in Figure 3.

#### 2.4.2. Hardened Properties

**Figure 4:**
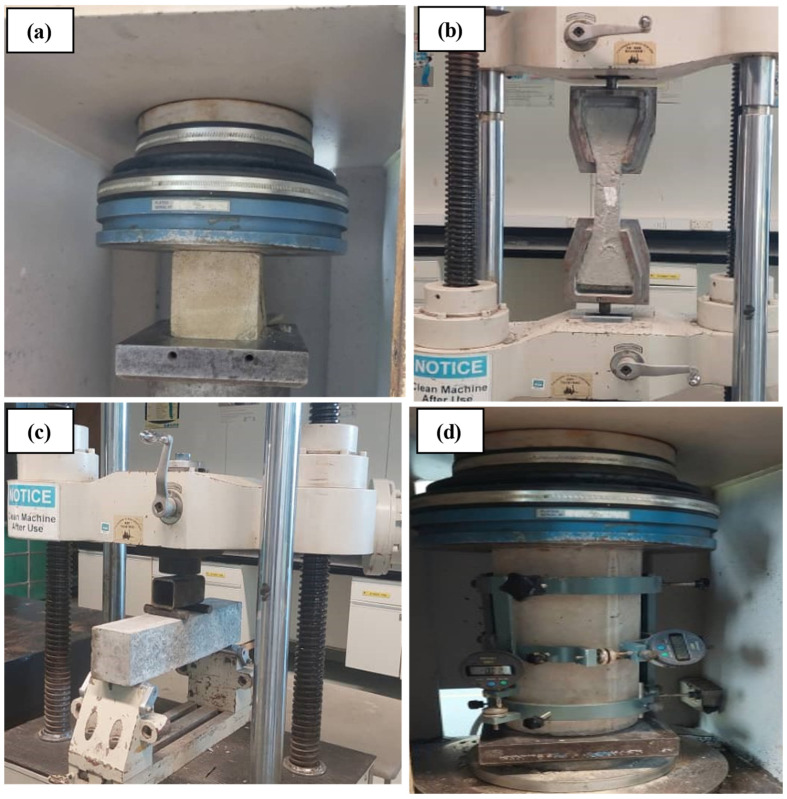
Experimental set-up for (**a**) CS, (**b**) TS, (**c**) FS, and (**d**) MOE and PR.

All the tests were performed at 28 days. The compressive strength (CS) was determined by following BS EN 12390-3 standard [45]. A four-point flexural strength (FS) test was executed in agreement with the necessities of BS EN 12390-5 [46]. A direct tensile test (TS) was carried out on dog-bone-shaped samples of 420 mm × 120 mm × 30 mm, as described in the JSCE [47]. The modulus of elasticity (MOE) and Poisson’s ratio (PR) were calculated utilizing the static technique in accordance with the ASTM C469 [48]. Each measurement was conducted on four cylinders (150 mm × 300 mm) and applied to ascertain the CS of the mixture; specifically, the maximum load and stress were noted. The remaining two cylinders were utilized to measure the PR and MOE. The experimental arrangement included a compressormeter fitted with dial readings connected to the specimen. These gauges were used to monitor the longitudinal and transverse distortions under a force equivalent to 40% of the optimum load determined from compressive testing. The experimental set up for the mechanical and deformation properties of CR concrete containing MWCNTs are shown in Figure 4.

## 3. Results and Discussions

### 3.1. Compressive, Tensile and Flexural Strengths

**Figure 5:**
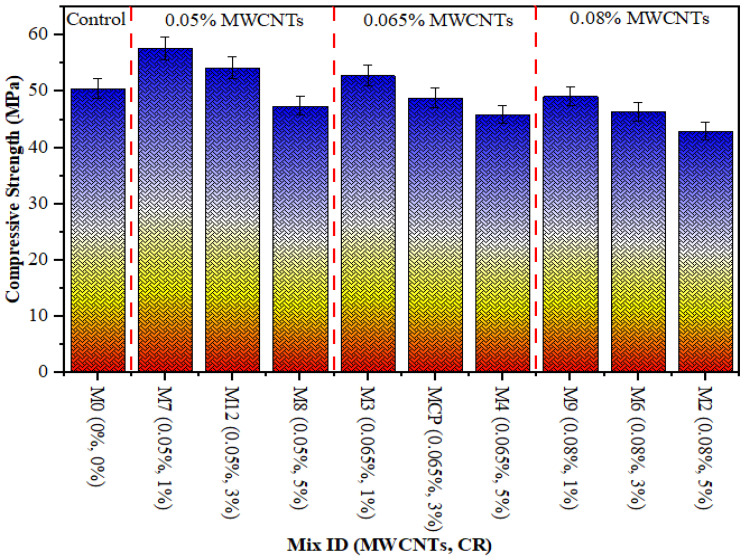
CS of rubberised concrete with MWCNTs.

**Figure 6:**
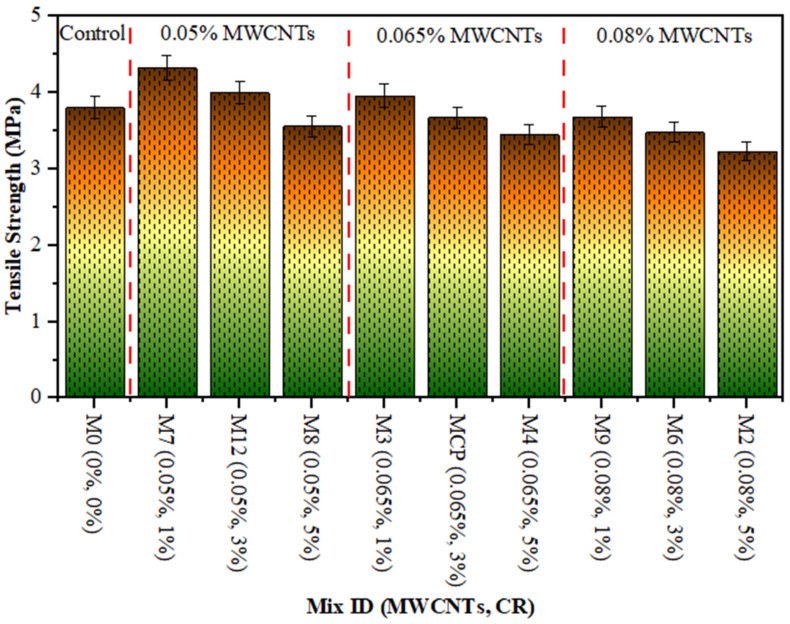
TS of rubberised concrete with MWCNTs.

**Figure 7:**
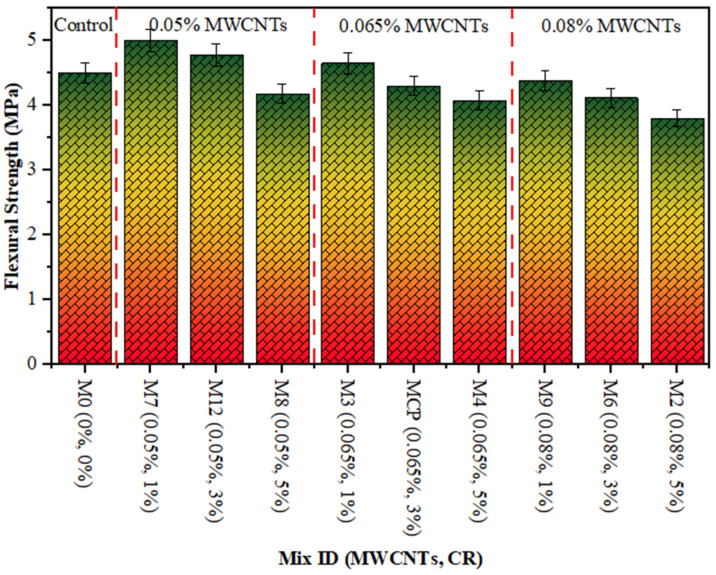
FS of rubberised concrete with MWCNTs.

Figure 5, Figure 6 and Figure 7 show the compressive, tensile and flexural strength results of CR concrete mixed with several amounts of MWCNTs at a curing age of 28 days, correspondingly. The maximum CS, TS, and FS were noted as 57.60 MPa, 4.32 MPa, and 5.0 MPa, respectively, for 0.05% of MWCNT and 1% of CR, while the lowest values were 42.97 MPa, 3.23 MPa, and 3.80 MPa, respectively, at 0.08% of MWCNT and 5% CR. The drop in strength with increasing CR is ascribed to the poor bonding between the hardened cement paste and CR particles. This is due to the hydrophobic nature of CR particles, which repel water and trap air on their surface, thereby thickening the ITZ. Furthermore, the softer texture and lower elastic modulus of CR, relative to sand particles, lead to a decrease in concrete strength. This occurs because the CR introduces weak points within the composite material. These observations are consistent with other studies that have confirmed the negative effect of adding more CR on concrete strength [41,42]. On the other hand, adding MWCNTs to the mixture significantly improves the microstructure of the hardened concrete, as the MWCNTs filling up the micro-voids leads to a denser and more uniform structure [49,50,51,52]. In addition, the enhancement in the strength of the rubberised mix is due to an excellent distribution of MWCNTs [53]. As MWCNTs accumulate in rubberised concrete, the strength decreases. Due to insufficient MWCNT distribution in the rubberised concrete matrix, CR concrete strength drops. Comparable findings was reported in other studies [53,54].

### 3.2. Modulus of Elasticity (MOE)

**Figure 8:**
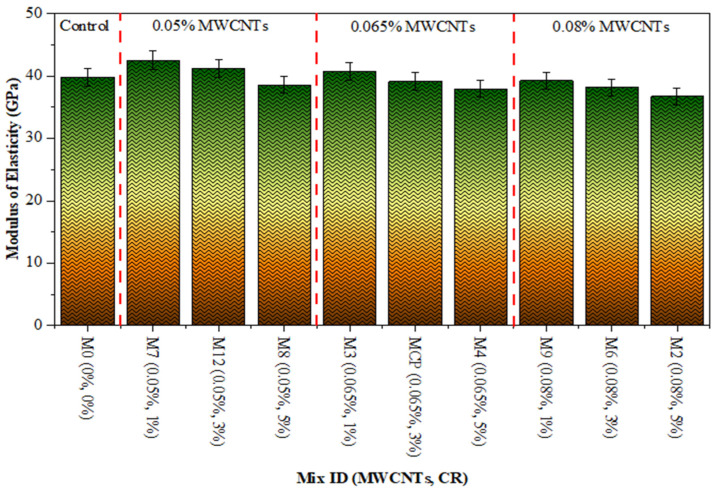
MOE of CR concrete blended with MWCNTs.

The MOE is a measurement utilized to quantify the capability of concrete to withstand distortion. It is a significant characteristic that is affected by several aspects, including the compatible nature of the building materials, the dimensions, form, and kind of aggregate, and ITZ [55]. The experiment has been performed in agreement with the necessities of ASTM C 469 [48]. The MOE of concrete depends significantly on its CS. Previously, scholars have proposed many equations to represent the relationship between different types of concrete CS and MOE. The development of these mathematical equations was based on empirical research [56]. A formula was derived to evaluate the MOE for CR concrete with varying water-to-cement ratios [57]. Figure 8 displays the MOE results for the thirteen runs of experiments. The MOE of CR concrete, mixed with different percentages of MWCNTs, was tested after 28 days. The maximum ME was recorded at 32.40 GPa using a combination of 0.05% MWCNTs and 1% CR. However, conditions of 0.08% MWCNTs and 5% CR resulted in the lowest ME of 27.53 GPa at 28 days. The findings indicate that as the CR replacement increased, there was a drop in the ME values for all the mixtures. This may be attributed to the reduced hardness of CR [58]. Nevertheless, there was a significant improvement noted due to the inclusion of MWCNTs in CR concrete. Using 0.05% exhibited a greater ME throughout all CR substitution levels. This improvement is attributed to the fineness of MWCNTs, which allows them to bridge microcracks, resulting in increased stiffness of the hardened CR concrete. In addition, the increases in ME may be accredited to a better relationship between the PC paste and aggregate, which leads to thickening of the ITZ [59,60]. According to prior research, the ITZ can enhance the MOE of the matrix [61,62]; therefore, the presence of MWCNT in the ITZ between the hardened cement paste and the CR surface plays a major role in thickening those ITZs, which leads to improved MOE values. However, 0.05% MWCNT in CR concrete caused the MOE to decline. This drop in MOE is due to MWCNT agglomeration. Attaining consistent dispersion of MWCNTs in CR concrete is a difficult task since it involves the movement of MWCNTs sliding against one another [63,64,65,66]. The outcomes were consistent with the findings of prior research [53,67].

### 3.3. Poisson’s Ratio (PR)

**Figure 9:**
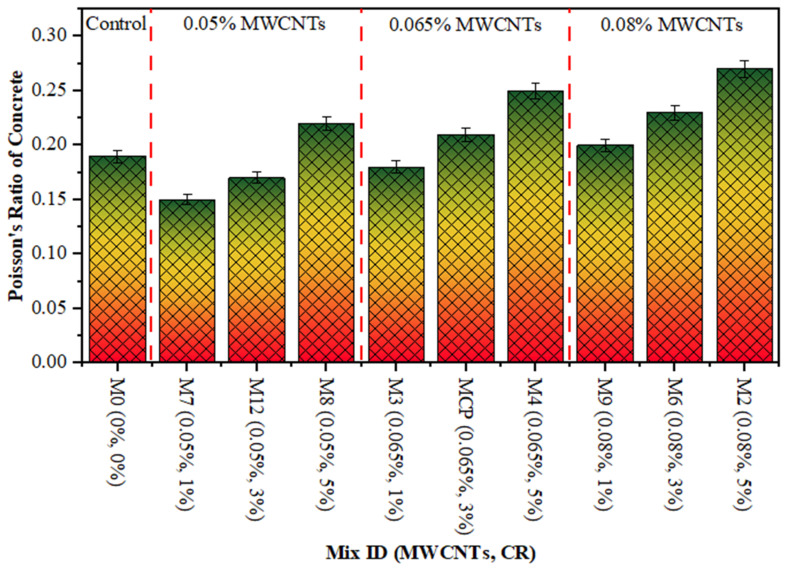
PR of CR concrete blended with MWCNTs.

The PR measures a specimen’s ability to deform by comparing the lateral strain to the longitudinal strain [68]. The static PR for cement-based concrete generally ranges from 0.15 to 0.25 [69]. Figure 9 illustrates that the PR ranged from 0.15 to 0.27 on 28 days across all specimens prepared with various amounts of CR and MWCNTs. The aforementioned significant values are consistent with the deformation characteristics of the composite material, which aligns with the findings of prior investigations into CR composites [70]. As the CR percentage increases in concrete, its ability to bend radially underneath stress also increases, leading to higher values of the PR. However, the PR decreased as the MWCNTs increased because of the filling effect of the MWCNTs when using up to 0.05% by PC weight, and this enhanced the stiffness of CR concrete. After the accumulation of 0.05% of MWCNTs in CR concrete, the PR decreased marginally owing to the improper dispersion of MWCNTs, which negatively impacted the CR concrete and reduced the toughness of the matrix. A similar observation was reported in prior research [70].

### 3.4. FESEM Analysis

**Figure 10:**
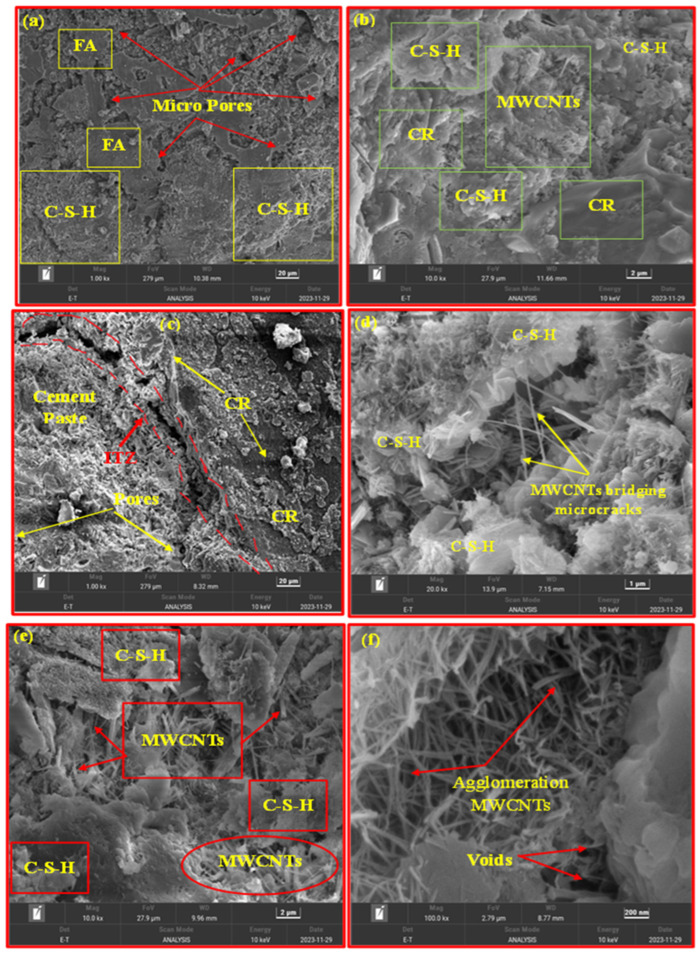
FESEM of rubberised concrete for (**a**) M0, (**b**) M7, (**c**) M6, (**d**) M9, (**e**) MCP, and (**f**) M2.

The mechanical properties of CR concrete were enhanced by the addition of MWCNTs in this investigation. To better understand this improvement process, rubberized concrete samples with different MWCNT % (0%, 0.05%, 0.065%, and 0.08%) were selected for microscopic property analysis. Figure 10a shows the presence of many pores in the control samples. Figure 10b,d,e depict the morphological characteristics of the surface of 1%, 1%, and 3% of CR in concrete blended with concentrations of 0.05%, 0.08%, and 0.065% MWCNTs, respectively. Nevertheless, MWCNTs were used to occupy the microscopic spaces of the cement matrix or to bridge microcracks, resulting in a more compact microscopic structure of the concrete. MWCNTs facilitated load transfer across microcracks, leading to improved mechanical characteristics in concrete. Several studies [29,71,72,73,74,75,76] have also confirmed these properties of CNTs with nano-scale benefits. Furthermore, the extraction and breakage of CNTs inside the cement matrix led to ineffective bridging phenomenon. This, in turn, absorbed the strain energy emitted by the fracture and hindered the advancement of the crack [77,78]. In addition, recent work also represents the fracture energy of concrete for paving applications under different modes to be absorbed through air-entrained cement paste, and the role of aggregates in blending was also found to be prominent [79,80,81]. Furthermore, the nucleation phenomenon plays a crucial role in enhancing the properties of cement-based substances reinforced with MWCNTs. Multiple sources suggest that MWCNTs can scatter cement particles, thereby offering more locations for the hydration of the cement reaction and hence speeding up the initial hydration reaction. Additionally, they help in densifying the microscopic structure and improving the crystallinity of materials, leading to increased CS [82,83,84]. Figure 10b,d show that MWCNTs act as nucleation sites, and are covered by C-S-H in the hydration process. This phenomenon exhibited consistency with the findings reported by other researchers [82,85].

Furthermore, the samples comprising CR exhibited a significant number of holes. CR’s participation led to an augmentation in the quantity of microscopic cracks throughout the concrete, as shown in Figure 10c. Figure 10c demonstrated that the hydrophobic nature of CR led to a considerable reduction in the ITZ strength of the concrete. Prior studies [73,86] have reported similar results. However, it was discovered that MWCNTs formed clusters inside the cement matrix and occupied empty spaces in the concrete sample with 0.08% MWCNT, as seen in Figure 10f. Comparable findings were identified in prior studies [67,75,87,88]. The inclusion of 0.08% MWCNT resulted in a reduction in strength. This outcome aligns with the results reported by Guan et al. [82]. However, this aids in comprehending the amalgamation impact and sway of CR and MWCNTs in the concrete composites. The presence of MWCNTs would reduce the amount of C-S-H covering on the external layer of cement particles as a result of the nucleation reaction [82]. Hence, water molecules have a higher propensity to interact with cement particles, speeding up dissolution, nucleation, and the formation of hydration products. Figure 10 demonstrates that the accumulation of MWCNTs leads to a reduction in the total volume of pores throughout the mixture, hence creating a compact hydration mechanism. The ITZ may become filled with products of hydration as a result of the insertion and formation of MWCNTs.

In addition, agglomerations of MWCNTs retain the ability to transmit stress and hinder the spread of cracks [67,89]. Higher CR may have an adverse effect on it. The negative consequences of rubber particles on concrete blended with MWCNTs are as outlined below: (i) The hydrophobic nature of CR leads to the creation of nearby regions with a low water–cement ratio, which decreases the interaction between the cement clinker and pore formation solution, thus slowing down the rate of hydration. (ii) The adsorption of MWCNTs onto the surface of the CR particles decreases the amount of MWCNTs that effectively take part in the nucleation process. Furthermore, the higher CR levels may stimulate the clumping and intertwining of MWCNTs, leading to a corresponding rise in the number of fragile bonds. (iii) The presence of CR hinders the ability of linked hydration products to interlock and bridge together due to its air-entraining impact. This might result in an increase in the separation between neighbouring nucleation sites, leading to the failure of some MWCNTs to form a bridge [67]. The MWCNTs system is capable of bridging a distance between neighbouring C-S-H clusters of 1 μm or less, but it is unable to cross bigger gaps [67,90].

The negative impact of 1% CR is comparatively less than that of a larger concentration. Consequently, a smaller number of small fractures are filled by the excess MWCNTs, and the reaction causing the MWCNTs to clump together is slightly less influenced by CR, or the complete filling of gaps caused by certain of the clumps offsets the decrease in strength. This might lead to the MWCNT0.05-CR1 achieving optimal strength at a macroscopic level. CR and MWCNTs are in direct competition as components in concrete composites. Increasing the rubber component to 5% increases the negative impact of CR, resulting in a further reduction in the optimal corresponding degree of MWCNTs to 0.05%. This suggests that the process of agglomeration and entanglement interactions takes place at an early stage in the concrete composite with 5% CR and 0.08% MWCNTs. The presence of an excessive amount of MWCNTs of 0.08% throughout the matrix adds to the weakening of the mechanism caused by rubber particles. Consequently, the rate of strength enhancement decreases as the content increases.

## 4. RSM Analysis

A comprehensive understanding of the RSM approach is crucial for comprehending the framework of the experimental design, such as the choice of input variables, their respective levels, and the nature of the experimental design (e.g., CCD or Box–Behnken). Moreover, the optimisation process, which often entails developing a mathematical model to depict the response as a function of the input factors, requires explanation. This includes the validation methods used for the model (e.g., ANOVA) and the identification of optimum circumstances, including the maximisation or minimisation of the response via desirability functions or other optimisation criteria. Understanding these processes would clarify the use of RSM and the derivation of the results.

### 4.1. Model Development and ANOVA

The response surface algorithms are constructed by applying linear, two factor interaction (2FI) and quadratic optimum predicted designs, which are indicated by Equations (1) and (2) [91,92] for linear and more advanced levels of polynomial equations, correspondingly.

(1) $$y=\beta_{0}+\beta_{1}x_{2}+\beta_{2}x_{2}+\beta_{n}x_{n}+\epsilon \;$$

(2) $$y=\beta_{0}+\sum_{i=1}^{k}\beta_{i}x_{i}+\sum_{i=1}^{k}\beta_{ii}x_{i}^{2}+\sum_{j=2}^{k}\sum_{i=1}^{j=1}\beta_{ij}x_{i}x_{j}+\epsilon \;$$

The variable y represents the response of concern. The linear, 2F and quadratic factors are denoted by *i* and *j*, correspondingly. The regression variable is represented by *β*. The variable *k* reflects the number of components that are being researched and optimized. The randomized error is denoted by the symbol ϵ.

The predicted response approaches were developed using the experimental results supplied in the RSM device, as demonstrated in Equations (3)–(7). The dependent parameters in this scenario are the CS, TS, FS, ME, and PR, which represent the responses. The independent parameters are denoted as *A* and *B* for the MWCNTs and CR, correspondingly, representing the input components. Using the selected sequential model sum of squares (SMSS), it was observed that a 2F model was highly suitable for the CS, TS and MOE variables, whereas linear models were used to match the slump, FS, and PR variables.

(3) CS=+49.39−3.45×A−3.88×B+1.01×AB

(4) TS=+3.70−0.25×A−0.29×B+0.077×AB

(5) FS=+4.35−0.28×A−0.38×B

(6) MOE=+29.81−1.15×A−1.29×B+0.30AB

(7) PR=+0.21−0.027×A+0.035×B

A comprehensive statistical analysis is crucial for validating the dependability of the constructed prediction models in conjunction with ANOVA. Essential metrics, including R^2^, corrected R^2^, and prediction error statistics, must be supplied to assess model accuracy. Residual analysis evaluates the hypotheses of normality and independence of errors, while cross-validation examines the model’s generalizability.

The ANOVA was performed on the derived algorithms with a reliability level of 95%. Therefore, all the variables that have a substantial impact on the replies contain a probability lower than 5% [93,94,95,96]. Likewise, all the algorithms exhibiting a possibility lower than 5% are considered meaningful. The ANOVA yielded significant results for the CS and TS approaches, specifically for variables *A*, *B*, and *AB*. These findings are demonstrated in Table 4. In the FS, MOE, and PR approaches, *A* and *B* were the significant factors, respectively. The lack of suitability is not statistically meaningful for all the mathematical models, but that is acceptable since it indicates a good match.

The design verification values shown in Table 5 indicate that all the constructed algorithms have a strong R^2^ varying from 93% to 99%. The R^2^, expressed as a percentage or a value between 0 and 1, indicates the degree of fit between the chosen model and the information. Greater R^2^ readings suggest a stronger fit for the model [41,97,98,99]. Therefore, in this case, all the constructed models accurately match the information. Furthermore, all the algorithms in this scenario meet the requirement that the difference between the adjusted and predicted R^2^ would not exceed 0.2, indicating their good performance. In addition, a sufficient level of accuracy is applied to quantify the ratio of signal to noise, and a minimum value of 4 is necessary. The accuracy ratings of the generated models vary from 28.22 to 60.13, all meeting the criteria for an appropriate signal.

### 4.2. Model Graphs and Diagnostic Plots

**Figure 11:**
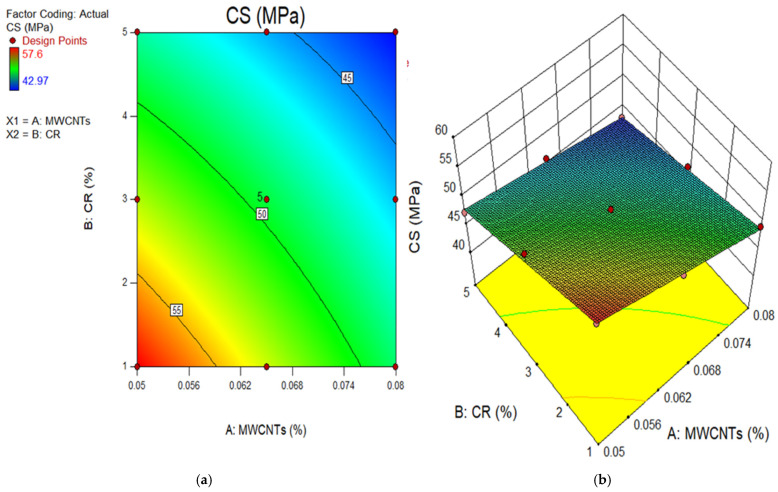
(**a**) 2D graph and (**b**) 3D Graph for CS.

**Figure 12:**
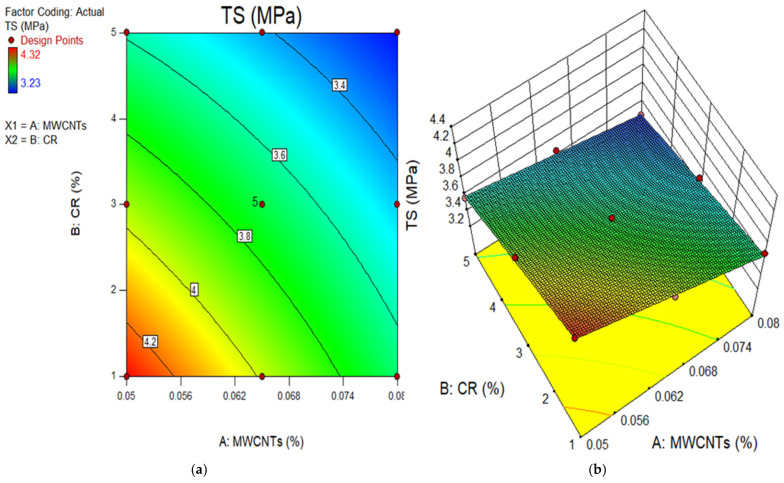
(**a**) 2D graph and (**b**) 3D graph for TS.

**Figure 13:**
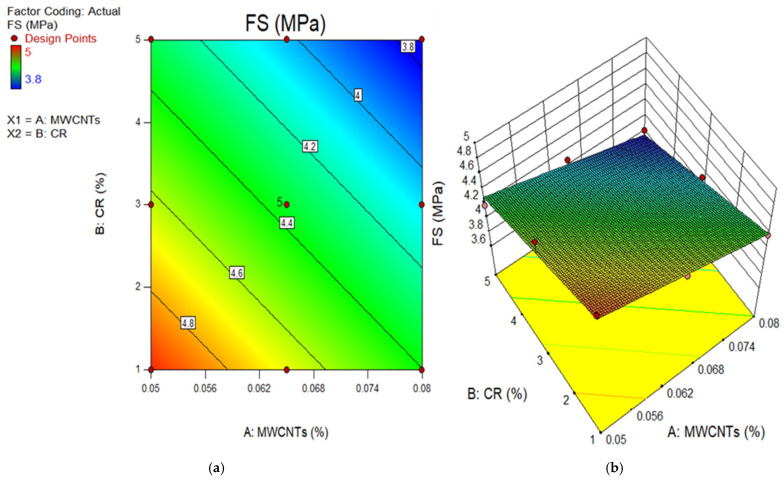
(**a**) 2D graph and (**b**) 3D graph for FS.

**Figure 14:**
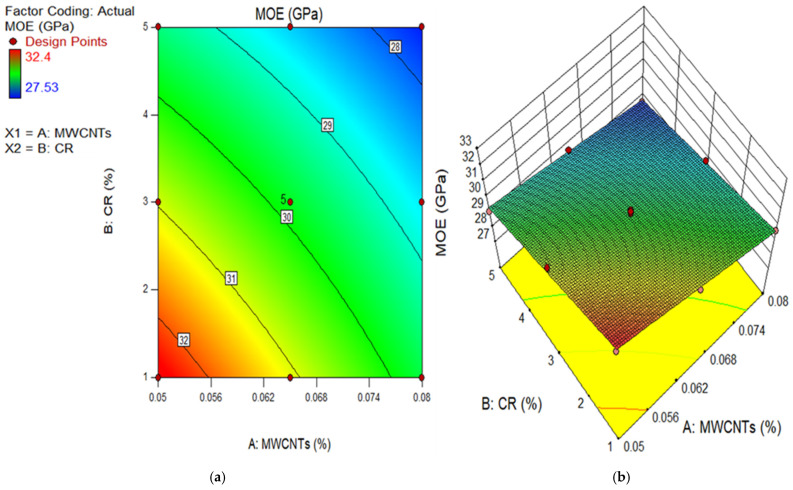
(**a**) 2D graph and (**b**) 3D graph for MOE.

**Figure 15:**
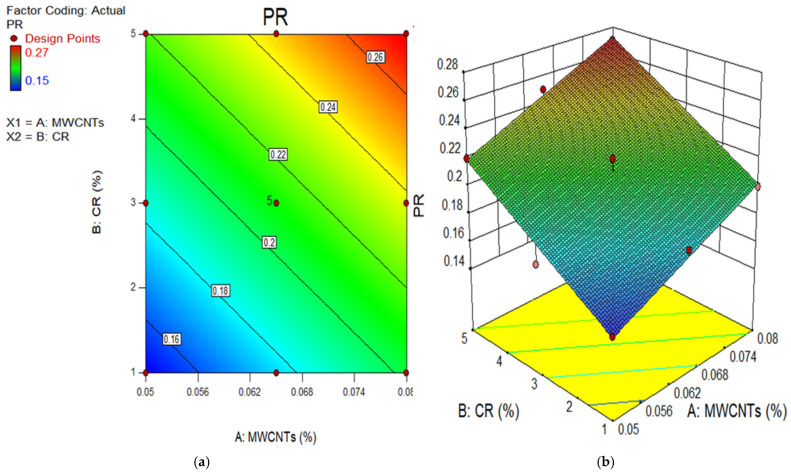
(**a**) 2D graph and (**b**) 3D graph for PR.

The 2D contour and 3D response surface graphs are used to depict the connection between the input factors and their individual relations, and their interaction effects on the dependent parameter. The plots depict the impact of independent factors on the outcomes using a colour gradient. The 2D figure illustrates the relationship between the parameters by displaying outlines that indicate different response degrees at certain values of the input components. In contrast, the 3D response surface graphs provide identical data to the 2D graphs, but in a three-dimensional structure, as indicated by the name. Figure 11, Figure 12, Figure 13, Figure 14 and Figure 15 show the 2D contour and 3D response surface graphs of the response algorithms that were built. The red areas in the graphs represent the greatest values of the outcomes, whereas the blue portions represent the smallest values. The yellow and green areas on the graphs indicate the medium range of the outcome.

Upon examining these design diagrams, it becomes evident that the influence of the input parameters (MWCNTs and CR) on the outputs aligns closely with the arguments presented in earlier sections. The 2D graph and 3D graph in Figure 11a,b, correspondingly, demonstrate the decrease in contours and gradual descent from the peak. This indicates that the CS significantly declined as the amount of CR increased, but it increased as the content of MWCNT rose. There is a transition from the red zone to the blue area on the diagrams. Therefore, in order to get a low CS value for the MWCNT-CR-concrete, it is necessary to increase the concentration of MWCNTs and CR. To get a high strength (above 55 MPa) in MWCNT-CR-concrete, it is recommended to employ 0.05% MWCNT at a concentration of 1% CR, as seen in Figure 11. Comparable explanations may be derived for the remaining design diagram.

**Figure 16:**
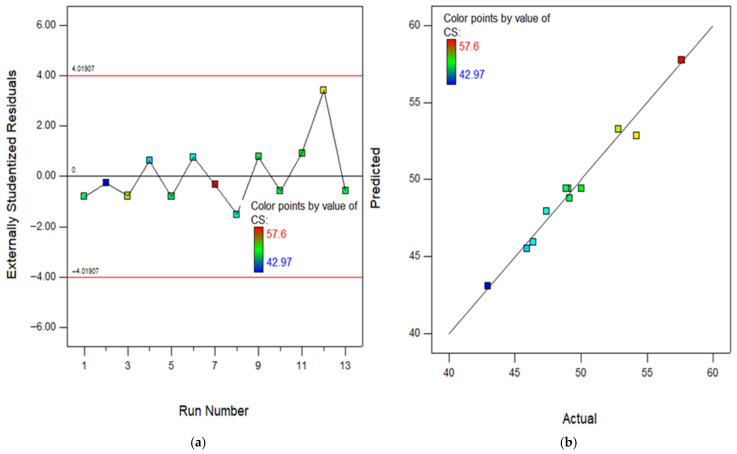
(**a**) Experimental runs against residual diagram and (**b**) actual results in contrast to predicted diagram for CS.

**Figure 17:**
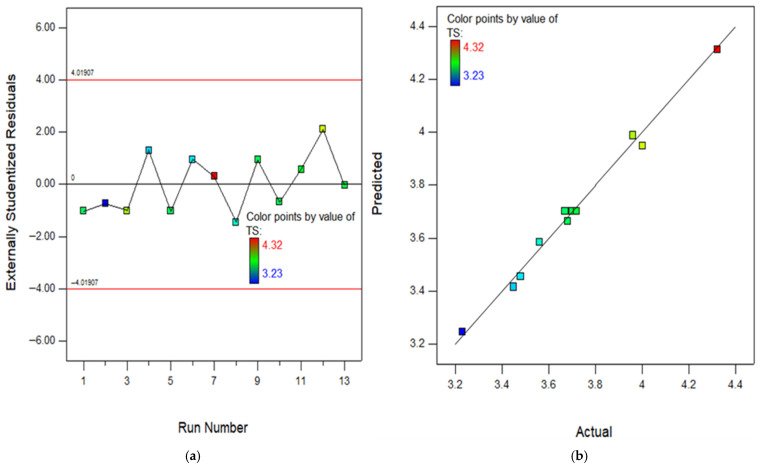
(**a**) Experimental runs against residual diagram and (**b**) actual results in contrast to predicted diagram for TS.

**Figure 18:**
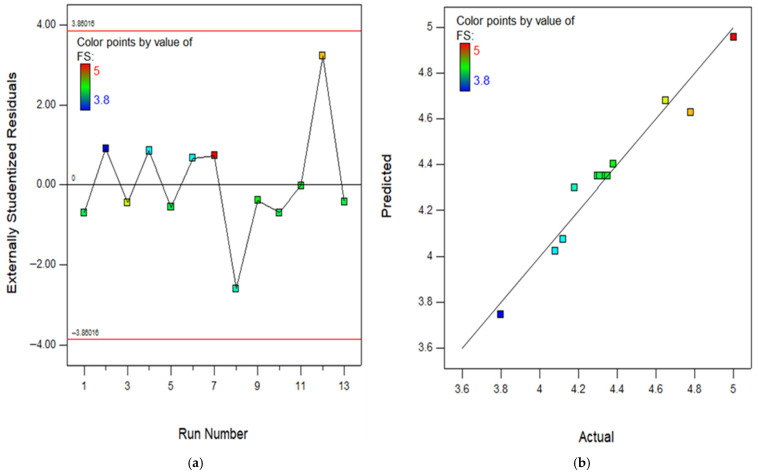
(**a**) Experimental runs against residual diagram and (**b**) actual results versus predicted diagram for FS.

**Figure 19:**
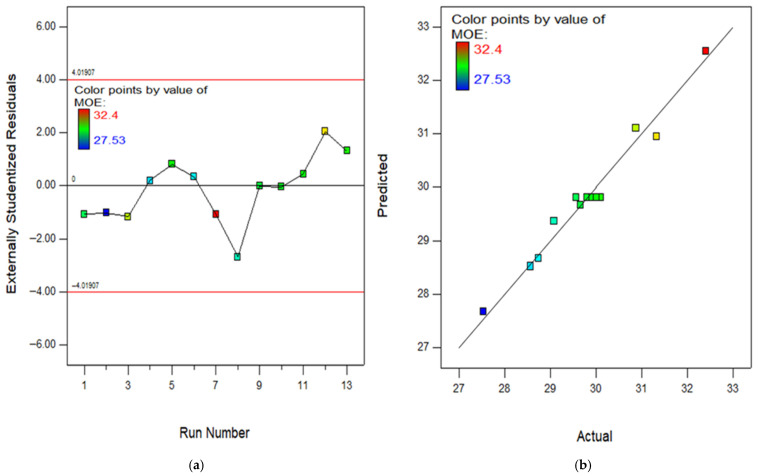
(**a**) Experimental runs against residual diagram and (**b**) actual results versus predicted chart for MOE.

**Figure 20:**
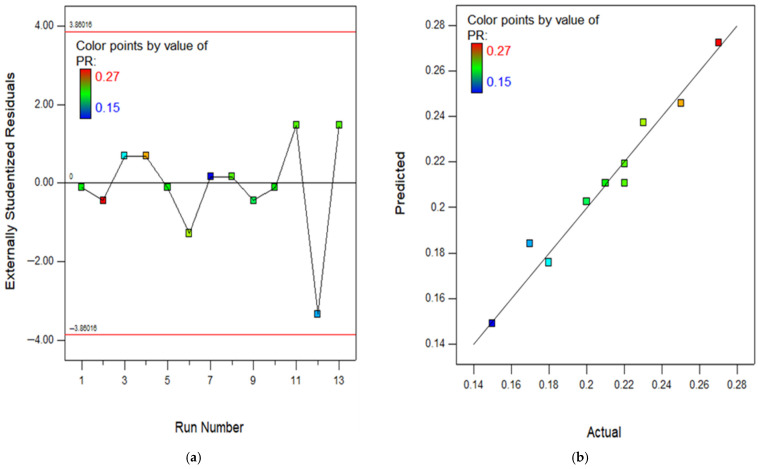
(**a**) Experimental runs against residual diagram and (**b**) actual results in contrast to predicted diagram for PR.

The RSM assessment includes many crucial diagrams, including the model diagnostic graphs. One of these graphs is the normal diagram of residuals, which consists of Figure 16, Figure 17, Figure 18, Figure 19 and Figure 20a showing the experimental runs against the predicted, and Figure 16, Figure 17, Figure 18, Figure 19 and Figure 20b showing the real results against the predicted. These diagrams demonstrate that the data is normally distributed along a straight line connecting the points. The closeness of a point to the line reflects the degree to which information corresponds to a standard distribution, whereas a larger distance implies the opposite. Based on the results, it is evident that the model is appropriate for the proposed application and might be utilized to establish the ideal obtaining parameters. The anticipated data for the model are developed by utilizing the prediction algorithm in the design expert programme, whereas the actual variables are achieved through experimental runs.

In addition, the diagram comparing the actual values to the projected values clearly evaluates the accuracy of the approach and displays the variability caused by random factors. The dispersion of the data points around a line of regression can be employed to measure the connection between the actual and anticipated outcomes, as seen in the scatter diagram. The proximity of the information values to the diagonal lines reflects the magnitude of the coefficient of determination of the approach. In order to get a perfectly suited approach, it is essential for the geographical distribution of all points to be uniform around the length of the line. The presence of skewness in the statistics will be evident when the points are mostly located on one side of the diagonal line. As seen in the (b) section of Figure 16, Figure 17, Figure 18, Figure 19 and Figure 20, all of the constructed models exhibit a strong fit, providing additional confirmation of their robustness and suitability for predicting responses.

### 4.3. Multi-Objective Optimization

Multi-objective optimisation is a field within various criteria-making choices that focuses on solving mathematical optimisation challenges in which many objective functions need to be optimised concurrently [100,101,102]. This technique is advisable since the majority of optimisation issues in practical scenarios include the identification of many optimal solutions among numerous conflicting goals. To get the desired outcomes without sacrificing the reactions, specific objectives are established for both the independent and dependent characteristics, taking into account different criteria and degrees of importance. The desirability value, denoted as dj, is applied to assess the optimisation outcome, and its value ranged from 0 to 1, as seen in Equation (8) [103]. The higher the result, the larger the dj value.

(8) $$D=(d_{1}^{r1}\times d_{2}^{r2}\times d_{3}^{r3}\times \cdots \cdots \cdots \cdots \cdots \cdots \cdots \cdots \cdots \times d_{n}^{rn}{)}^{\frac{1}{n}}\;$$

The variable *n* denotes the number of responses measured in the optimisation procedure, whereas *ri* represents the importance degree of every objective variable di.

**Figure 21:**
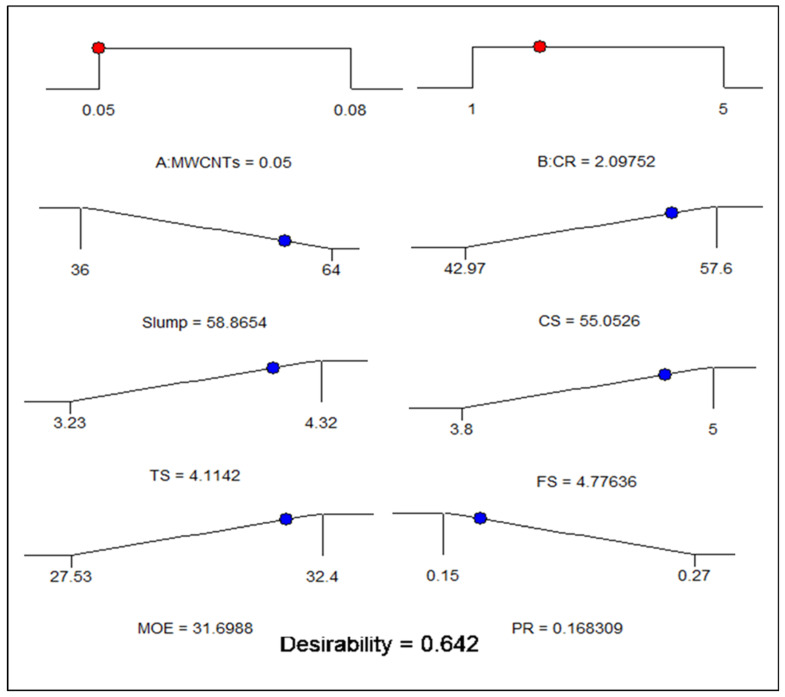
Ramp solutions for optimization of CR concrete.

**Figure 22:**
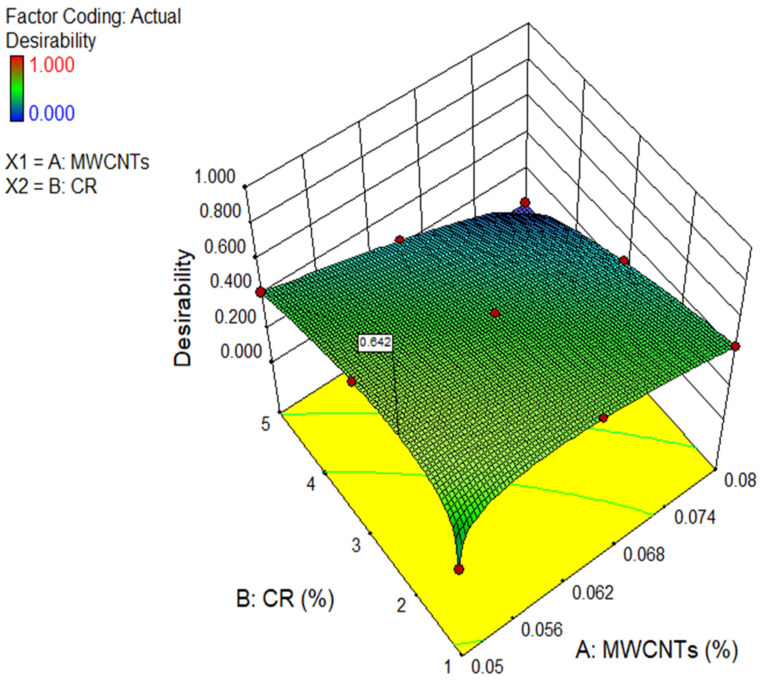
3D diagram of desirability of CR concrete.

The goal parameters for the input and output elements are shown in Table 6. To get the most favourable outcomes with the least amount of MWCNT and CR possible, the strategy was to minimise the MWCNTs and CR. The MWCNTs and CR were selected throughout the range of frequencies used in the investigation. The CS, TS, FS, and MOE of concrete were optimised, while the PR was minimised. Among the five accessible phases, 03 (the default) was maintained as the primary level for all the elements. The resultant solution after running the optimisation shown in Figure 21 as a series of ramps. The first outcome offered by the RSM was selected out of the nine options available. The most effective input parameters were 0.05% of MWCNT and 2.09% of CR. These variables resulted in the greatest values for CS, TS, FS, MOE, and PR, which were 55.05 MPa, 4.11 MPa, 4.77 MPa, 31.69 GPa, and 0.168, correspondingly. The overall desirability of these results was 0.642. Figure 22 displays a three-dimensional graphic illustrating the desirability of the optimisation process.

### 4.4. Investigational Validation

The last stage of the RSM evaluation covers a practical validation of the developed response prediction algorithms. This was achieved by manufacturing specimens consisting of concrete blended with MWCNTs and CR material obtained from the optimized mixture. Three concrete examples were fabricated, utilising the best combination to figure out the CS, TS, FS, MOE, and PR. After a 28-day period of curing, the samples were subjected to a number of tests. The mean laboratory results, together with the anticipated consequences, are shown in Table 7. Equation (9) computes the difference between the observed and predictable values of each response. The findings show a high level of consensus across all concrete characteristics, with a difference value below 6%. This determines that the pre-existing response-predicting algorithms are reliable and accurate.

(9) $$\delta =\left|\frac{\vartheta_{E}-\vartheta_{P}}{\vartheta_{P}}\right|\times 100\%$$

Here,

δ denotes the percentages error (%);

$\vartheta_{E}$ represents the experimental outcomes;

$\vartheta_{P}$ shows the predicted outcomes.

## 5. Conclusions

The following key points are drawn from this research work:

- The incorporation of CR into concrete reduces its strength, while the use of MWCNTs leads to improvements to the mechanical properties of CR concrete. The highest CS, TS, and FS were recorded at 57.60 MPa, 4.32 MPa, and 5.0 MPa in rubberized concrete after 28 days. The optimal doses for MWCNT in combination with CR were consistently found to be 0.05% and 1%, respectively.
- The enhancements in the strength of the MWCNT–CR concrete may be attributed to the even distribution of MWCNTs, which act as nucleation sites for the formation of C-S-H. Due to its extensive surface area, the nanoscale enhances the speed of hydration.
- The highest value of ME was measured at 42.50 GPa while using a mixture of 0.05% MWCNT and 1% CR after 28 days. As the CR replacement level increased, there was a corresponding drop in the MOE for all the mixtures. However, using MWCNTs will mitigate this reduction.
- The Poisson’s ratio ranged from 0.15 to 0.27 over 28 days across all specimens prepared with various amounts of CR and MWCNTs. For MWCNT–CR concrete, the PR values remained within the acceptable range of 0.15 to 0.28.
- FESEM results revealed an improved and more precise microstructure when using MWCNTs in CR concrete. This is attributed to the pore-filling ability and crystallization of hydration products by the MWCNTs, resulting in a pore-refining action.
- ANOVA and experimentation served to create and validate response-predictive models. The constructed models had high R^2^ values, fluctuating from 93 to 99%. The multi-objective optimization yielded optimal input parameters of 0.05% and 2.09% for MWCNTs and CR, correspondingly, with a desirability value of 0.642.
- It is observed from the optimization through RSM modelling that the optimum mix (0.05% of MWCNTs and 2.09% of CR) can be used for practical application in real-world construction, and we recommended this as the optimum mix for field implementation.
- The utilization of the optimum mix in construction can reduce the cost of the project, reduce the environmental concerns, and utilize waste material (CR) for construction purpose.

## Figures and Tables

**Table 1:** Chemical compositions of PC.

Materials	Compound (%)									Specific Gravity	Blaine Fineness (m^2^/Kg)	Loss on Ignition
	SiO_2_	Fe_2_O_3_	Al_2_O_3_	CaO	MnO	Na_2_O	MgO	T_2_O	K_2_O			
PC	20.76	3.35	5.54	61.4	-	0.19	2.48	-	0.78	3.15	325	2.20

**Table 2:** Properties of MWCNTs [41].

Properties	Length (µm)	Size (nm)	Surface Area (m^2^/g)	No: of Walls	Purity (%)	Modulus of Elasticity (TPa)	Tensile Strength (GPa)	pH
**Values**	0.1–10	5–15	250–300	3–15	>95	1.28	100	4–10

**Table 3:** Mix design of rubberised concrete containing MWCNTs.

Mix ID			Materials				
	MWCNTs (%)	CR (%)	PC (kg/m^3^)	CA (kg/m^3^)	CR (kg/m^3^)	Sand (kg/m^3^)	Water (kg/m^3^)
M0	0.00	0	575	1425	0	662	202
M1	0.065	3	575	1425	13.11	648.89	202
M2	0.08	5	575	1425	21.85	640.15	202
M3	0.065	1	575	1425	4.36	657.64	202
M4	0.065	5	575	1425	21.85	640.15	202
M5	0.065	3	575	1425	13.11	648.89	202
M6	0.08	3	575	1425	13.11	648.89	202
M7	0.05	1	575	1425	4.36	657.64	202
M8	0.05	5	575	1425	21.85	640.15	202
M9	0.08	1	575	1425	4.36	657.64	202
M10	0.065	3	575	1425	13.11	648.89	202
M11	0.065	3	575	1425	13.11	648.89	202
M12	0.05	3	575	1425	13.11	648.89	202
M13	0.065	3	575	1425	13.11	648.89	202

**Table 4:** ANOVA outcomes.

Response	Source	Sum of Squares	Df	Mean Square	F-Value	*p*-Value > F	Significance
	Model	165.75	3	55.25	120.55	<0.0001	Yes
	A-MWCNTs	71.55	1	71.55	156.13	<0.0001	Yes
	B-CR	90.09	1	90.09	196.58	<0.0001	Yes
	AB	4.10	1	4.10	8.95	0.0152	Yes
Compressive Strength	Residual	4.12	9	0.46			Yes
	Lack of Fit	3.19	5	0.64	2.73	0.1763	Not
	Pure Error	0.94	4	0.23			
	Cor Total	169.87	12				
	Model	0.89	3	0.30	287.37	<0.0001	Yes
	A-MWCNTs	0.37	1	0.37	359.59	<0.0001	Yes
	B-CR	0.49	1	0.49	479.18	<0.0001	Yes
	AB	0.024	1	0.024	23.35	0.0009	Yes
Tensile Strength	Residual	9.261 × 10^3^	9	1.029 × 10^3^			Yes
	Lack of Fit	7.381 × 10^3^	5	1.476 × 10^3^	3.14	0.1451	Not
	Pure Error	1.880 × 10^3^	4	4.700 × 10^4^			
	Cor Total	0.90	12				
	Model	1.11	2	0.55	97.22	<0.0001	Yes
	A-MWCNTs	0.46	1	0.46	80.73	<0.0001	Yes
	B-CR	0.65	1	0.65	113.70	<0.0001	Yes
Flexural Strength	Residual	0.057	10	5.689 × 10^3^			Yes
	Lack of Fit	0.055	6	9.194 × 10^3^	21.38	0.0053	Yes
	Pure Error	1.720 × 10^3^	4	4.300 × 10^4^			
	Cor Total	1.16	12				
	Model	18.26	3	6.09	108.13	<0.0001	Yes
	A-MWCNTs	7.87	1	7.87	139.77	<0.0001	Yes
	B-CR	10.04	1	10.04	178.32	<0.0001	Yes
	AB	0.35	1	0.35	6.29	0.0334	Yes
Modulus of Elasticity	Residual	0.51	9	0.056			
	Lack of Fit	0.34	5	0.068	1.63	0.3286	No
	Pure Error	0.17	4	0.042			
	Cor Total	18.76	12				
	Model	0.012	2	5.808 × 10^3^	122.12	<0.0001	Yes
	A-MWCNTs	4.267 × 10^3^	1	4.267 × 10^3^	89.70	<0.0001	Yes
	B-CR	7.350 × 10^3^	1	7.350 × 10^3^	154.53	<0.0001	Yes
Poisson’s Ratio	Residual	4.756 × 10^4^	10	4.756 × 10^5^			
	Lack of Fit	3.556 × 10^4^	6	5.927 × 10^5^	1.98	0.2656	No
	Pure Error	1.200 × 10^4^	4	3.000 × 10^5^			
	Cor Total	0.012	12				

**Table 5:** Model verification constraints.

Model Validation Constraints	CS	TS	FS	MOE	PR
Standard Deviation	0.68	0.032	0.075	0.24	6.897 × 10^3^
Mean	49.39	3.70	4.35	29.81	0.21
C.V. %	1.37	0.87	1.73	0.80	3.27
PRESS	9.56	0.023	0.12	1.63	7.476 × 10^4^
−2 Log Likelihood	21.97	−57.32	−33.72	−5.29	−95.91
R^2^	0.9757	0.9897	0.9511	0.9730	0.9607
Adj R^2^	0.9676	0.9862	0.9413	0.9640	0.9528
Pred R^2^	0.9437	0.9744	0.8994	0.9132	0.9382
Adeq Precision	39.030	60.134	33.396	37.058	37.226

**Table 6:** Parameter optimization.

Factors	Input Factors					Responses (Output Factors)		
	MWCNTs (%)		CR (%)	CS (MPa)	TS (MPa)	FS (MPa)	MOE (GPa)	PR
Value	Minimum	0.05	1	42.97	3.23	3.80	27.53	0.15
	Maximum	0.08	5	57.60	4.32	5.00	32.40	0.27
Goal		Range	Range	Max.	Max.	Max.	Max.	Min.
Optimization Results		0.05	2.09	55.05	4.11	4.77	31.69	0.168
Desirability					0.642 (64.20%)			

**Table 7:** Optimization of investigational validation.

Responses	Practical Outcomes	Predicted Outcomes	Error (%)
CS (MPa)	56.28	55.05	2.23
TS (MPa)	4.20	4.11	2.18
FS (MPa)	4.92	4.77	3.15
MOE (GPa)	30.00	31.69	5.63
PR	0.16	0.168	4.78

## Data Availability

The original contributions presented in this study are included in the article. Further inquiries can be directed to the corresponding author.
